# From Stochastic Foam to Designed Structure: Balancing Cost and Performance of Cellular Metals

**DOI:** 10.3390/ma10080922

**Published:** 2017-08-08

**Authors:** Dirk Lehmhus, Matej Vesenjak, Sven de Schampheleire, Thomas Fiedler

**Affiliations:** 1 ISIS Sensorial Materials Scientific Centre, University of Bremen, 28359 Bremen, Germany; 2 MAPEX Center for Materials and Processes, University of Bremen, 28359 Bremen, Germany; 3 Faculty of Mechanical Engineering, University of Maribor, 2000 Maribor, Slovenia; matej.vesenjak@um.si; 4 Department of Flow, Heat and Combustion Mechanics, Ghent University, 9000 Ghent, Belgium; Sven.DeSchampheleire@UGent.be; 5 School of Engineering, The University of Newcastle, Callaghan NSW 2308, Australia; Thomas.Fiedler@newcastle.edu.au

**Keywords:** cellular metal, metallic foam, metal foam, porous materials, lattice materials, costs, manufacturing, additive manufacturing, mechanical properties, energy absorption

## Abstract

Over the past two decades, a large number of metallic foams have been developed. In recent years research on this multi-functional material class has further intensified. However, despite their unique properties only a limited number of large-scale applications have emerged. One important reason for this sluggish uptake is their high cost. Many cellular metals require expensive raw materials, complex manufacturing procedures, or a combination thereof. Some attempts have been made to decrease costs by introducing novel foams based on cheaper components and new manufacturing procedures. However, this has often yielded materials with unreliable properties that inhibit utilization of their full potential. The resulting balance between cost and performance of cellular metals is probed in this editorial, which attempts to consider cost not in absolute figures, but in relation to performance. To approach such a distinction, an alternative classification of cellular metals is suggested which centers on structural aspects and the effort of realizing them. The range thus covered extends from fully stochastic foams to cellular structures designed-to-purpose.

## 1. Introduction

According to generally accepted knowledge, the history of cellular metals started in the 1920s with a French patent [1], which, like two American ones published roughly 20 years later [2,3], only attracted limited attention. Cellular metals experienced their first boost in interest in the late 1950s and 1960s, again based on a row of US patents filed by Elliot [4,5,6], but they did not really start to fly until the early 1990s. Around this time, patents by Baumeister [7,8] met with a fertile environment that looked for new options in lightweight design and spawned development efforts on a broader scale which ultimately led to the vast variety of cellular metals we have at hand today. This diversity of materials and processes has been comprehensively discussed by authors like Banhart [9,10] and Lefebvre et al. [11], as well as Duarte et al., who focused on composite metal foams in a contribution to the current special issue [12].

It is a commonplace observation that major interest among the research community does not necessarily go with immediate introduction in industrial environments. Cellular metals are no exception in this respect and, especially in the 1960s, originally very high expectations concerning the new material were disappointed in the end. In a way, the boundary conditions that ruled these materials’ markets proved not to be favorable enough for wider acceptance at that specific moment in time. In the 1990s, the situation had changed: to name but one example, environmental concerns had raised interest in lightweight design in the transport industry as means towards reduced fuel consumption [13]. This heightened level of attention helped to channel research funds towards metal foam-related projects, which in turn led to improvement of materials, processes, and, consequently, performance—all this against the background of a starting point which, thanks to process improvements introduced in the 1990s, had improved in comparison to the 1960s.

With cost lowered, performance improved, and a strong demand for lightweight materials, three factors came together that all favored further metal foam development. Nevertheless, metal foams did not suddenly turn into cheap materials, and closer investigation revealed that in a number of application scenarios, the market demanded other solutions offering higher performance, even if it came at higher cost. One such example is aircraft floor panels. Different studies [14] have investigated the suitability of metal foams and metal foam-based sandwich materials for this area of application, but based on a better performance-to-weight ratio in this specific scenario, honeycomb-based solutions have prevailed in this application despite their higher cost. Basically, this case study illustrates that the question of whether a specific material or structure is economically most viable for a specific application is not governed by performance and cost alone. Even the definition of cost of performance as a new indicator linking economic and technological aspects does not suffice to determine which material to choose for a given use case, because it only describes the supply side of the problem and neglects demand. If the latter is pressing enough, higher cost will be accepted if the financial return associated with performance increase creates an overall economic advantage. Lightweight design in the transport industry is a graphic example in this respect. In the automotive industry, a weight reduction of 100 kg is usually linked to a fuel consumption lowered by 0.2 to 0.6 liters per 100 km. Assuming a service life of 200,000 km, this translates to some 400 to 1200 liters of fuel saved, or 4 to 12 liters per single kg. For a typical passenger aircraft’s service life of 20 years, a comparable calculation based on data published by Lufthansa for an Airbus A340-600 long haul aircraft leads to 1300 liters of fuel saved per kg of weight reduction. This underlines the much greater economic impact of weight reduction in aerospace applications and thus supports the aforementioned decision favoring honeycomb-based floor panels [15]. 

However, despite these reservations, would cost of performance be available for different materials, matching enhanced capabilities with requirements, and, in doing so, including the economic side of the problem would be simplified significantly. Providing basic data, as well as suggesting first approaches of this kind, is our intention with the present paper. We will study different types of cellular metals and consider particularly how added efforts in achieving structural control are reflected in costs, and how these balance with the gain in performance achieved thus. To limit the scope of this paper, we have chosen to restrict our study to mechanical properties, and here specifically to (density-related) compressive strength as well as energy absorption capabilities.

**Figure 1:**
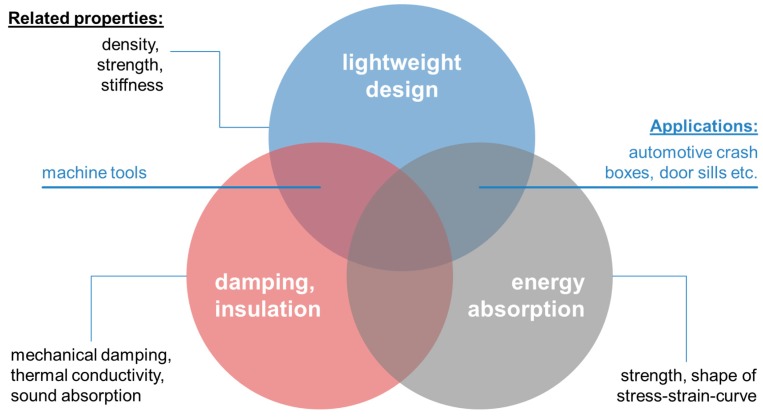
Characteristic property domains of metal foams and associated application scenarios, the latter based on information gathered by Garcia-Moreno and Weise [14,16]. Property domain representation inspired by Banhart [9].

The background is defined by the variety of available cellular metals as well as their applications. An up-to-date review of the latter has been provided by Garcia-Moreno as part of the present special issue [16]. This paper clearly shows that metal foams have indeed found widespread application even in scenarios that require larger scale production, like the use of façade materials in architectural design. Interestingly, though not surprisingly, a closer scrutiny of the case studies gathered in this work underlines the notion that metal foams are typically applied whenever they can bring in their unique combination of properties. Usually, it is this rather than excellence in a single field like weight-specific stiffness which makes the difference. This insight has already been highlighted in the past by several authors and is reflected in Figure 1, which in parallel attempts to position some of the applications collected by Garcia-Moreno in relation to the property domains stressed. Of course, the image can be expanded into further dimensions by including functional properties. We have refrained from doing so here since our survey is focused on mechanics, too.

Performance improvement is the complementary approach to cost reduction when it comes to enhancing economic viability of cellular metals. Much more than cost reduction, this path has been the primary course of research on cellular metals in the past, besides understanding of the foam formation itself in the case of actual metal foams. Naturally, the meaning of the term ‘performance’ is closely linked to a particular application and thus difficult to generalize. However, the following properties, or combinations of these, are crucial for the majority of uses:

- Mechanical Properties
  ○ Material stiffness (e.g., Young’s modulus): structural applications
  ○ Material strength
    ■ X% offset yield stress: structural applications
    ■ Plateau stress (magnitude and stability): energy absorption
  ○ Mechanical damping: structural and functional (e.g., damping elements) applications
  ○ Designed mechanical characteristics like anisotropy, auxetic behavior: diverse applications.
- Functional Properties
  ○ Thermal conductivity: thermal energy storage and conduction
  ○ Flow resistance: heat exchangers
  ○ Electrical conductivity: electrodes
  ○ Acoustic damping: sound absorption
  ○ Internal surface area: thermal applications, catalysts.

Looking primarily at mechanical properties, from a practical point of view, there are at least four different, generic ways of enhancing cellular metal and metallic foam properties. These are:

1. Raising property levels via the matrix material, e.g., through:
  ○ choice of higher strength matrix materials (e.g., [17,18])
  ○ heat treatment of matrix materials (e.g., [17,19,20])
  ○ reinforcement of matrix materials, composite matrices (e.g., [21])
2. Raising property levels via structural features, e.g., through:
  ○ material and/or process adaptations improving structural control
    ■ blowing agent pre-treatment (e.g., [22,23,24])
    ■ modification of surface tension and/or viscosity through ceramic particle addition (e.g., [21,25,26])
    ■ viscosity adaptation/control (e.g., Ca addition in Alporas^®^ foam, [27,28])
  ○ alternative processes with inherently better control of/less freedom in structure development, such as,
    ■ GASAR [29,30] and lotus type foams [31,32,33,34]
    ■ syntactic foams [35,36,37,38,39,40]
    ■ assembled structures like Kagome and wire-based materials [41]
    ■ designed structures realized via additive manufacturing [42]
3. Lowering the scatter of properties to allow for reduction of safety factors, thus increasing the practically accessible material properties, as done in
  ○ APM foams [43,44,45,46]
  ○ metal hollow sphere-based foams [47,48]
  ○ syntactic foams [49]
  ○ UniPore structures [50,51,52]
4. Use of metal foams as one of several components in multi-material, hybrid, and similar structures
  ○ integral foams
    ■ integral foam casting [53,54,55]
    ■ integration of foam parts in metal castings
    ■ two component processes for syntactic foams produced via metal injection molding (MIM) [56]
  ○ sandwich structures
    ■ Aluminium Foam Sandwich (AFS) [10,57]
    ■ steel face sheet sandwich materials via foaming between face sheets [58]
    ■ APM-based sandwich [59]
    ■ CFRP-metal foam sandwich structures
    ■ etc.
  ○ foam-filled hollow components
    ■ in situ foaming to achieve foam-filled tubes and extrusions [60,61,62,63]
    ■ co-extrusion of conventional and foamable material followed by in situ foaming [8]
    ■ ex situ foaming and subsequent adhesive bonding of cores to hollow metallic structures [61,64,65]
    ■ ex situ foaming and subsequent adhesive bonding of cores to hollow CFRP structures, including use of metal foam parts as permanent core/mandrel in FRP production, e.g., filament winding [66,67]
    ■ etc.
  ○ hybrid foams
    ■ Polymer foam-APM hybrids [68,69]
    ■ etc.

The implications of such techniques on cellular metal performance have been widely studied. In some cases, specifically where a supporting use of cellular metals (e.g., in hollow structures or sandwich cores) is foreseen, separating the cellular metal’s contribution from the structure’s integral characteristics becomes difficult, since part performance is synergistically derived from the individual components’ characteristics—the whole proverbially being more than the sum of its parts in these cases. Similarly, the cellular metal’s contribution to cost is hard to grasp if raw materials as well as processing are to be covered. For this reason, when looking at costs, and beyond it at cost of performance, we will concentrate on the actual cellular metals and deliberately exclude structural solutions relying on them as one component among others. Effectively, this point of view rules out most of the above listing’s option 4, except for those cases where hybridization takes place on a material rather than a structural level, i.e., the so-called hybrid foams.

In contrast to their performance, the actual cost of metal foams has rarely been the primary object of study, even though cost reduction has been named as motivation in several investigations found in the scientific literature. The following list offers an overview of the different perspectives assumed in these and names handles that have been considered in the past in view of their cost reduction potential. The starting point is the situation at the beginning of the 1990s, and thus the powder compact melting process which initiated the metal foam renaissance [7,8].

- replacement of powders by metallic melts (e.g., FORMGRIP and FOAMCARP process, [70,71])
- replacement of powders with lower-cost particulate materials like machining chips (e.g., [72,73])
- replacement of costly blowing agents like titanium hydride with lower-cost variants (e.g., calcium carbonate and/or dolomite (e.g., FOAMCARP process [71,73])
- replacement of costly blowing agents by inexpensive porous filler particles (e.g., [38,74,75])
- separating the part shaping and the metal foaming/primary porosity creation process (e.g., hollow sphere based structures [47], APM foams [43])
- process automation to minimize cost-intensive manual labor.

**Figure 2:**
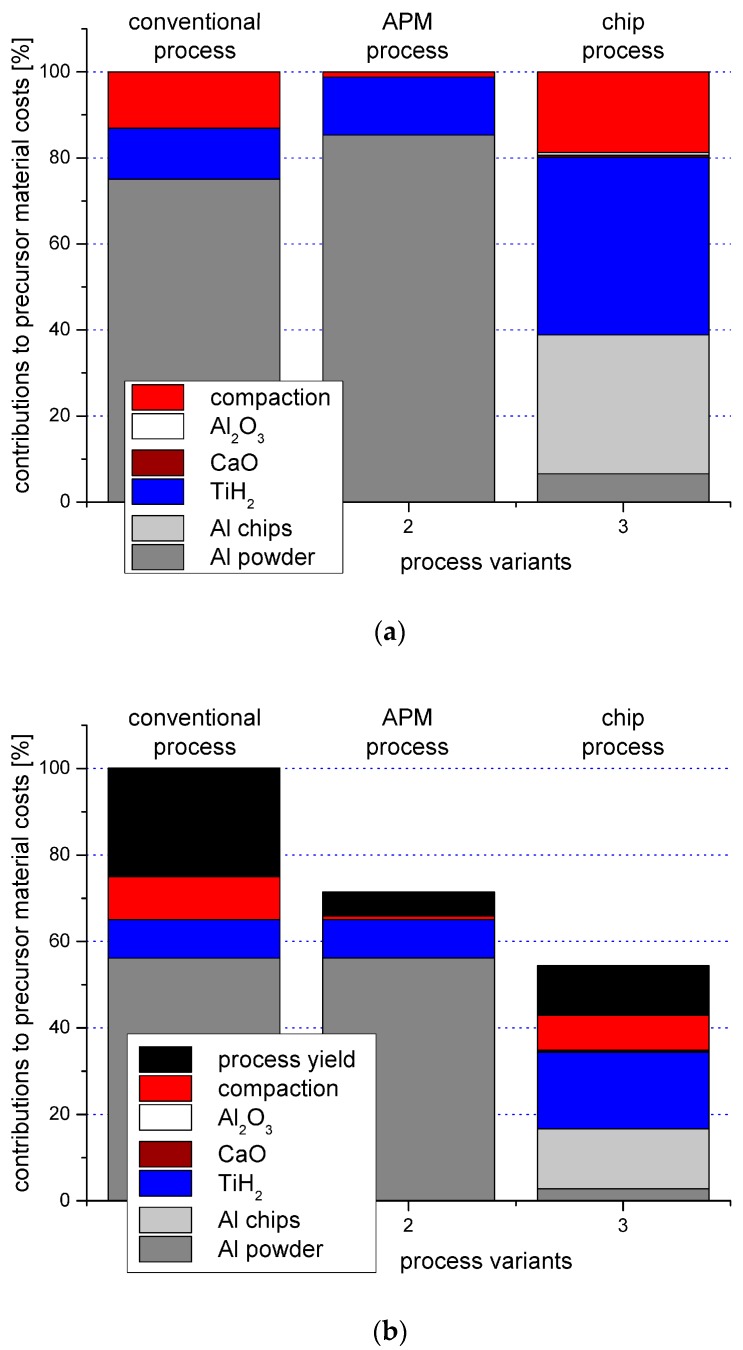
Evaluation of precursor material costs as performed by Lehmhus et al.: Top, contributing factors excluding process yield; bottom, cost reduction potential relative to the conventional powder compact melting or Foaminal™ process [72].

The effect of some of these measures on cost has been studied by Lehmhus et al., who compared powder and chip-based aluminum foams using different types of blowing agents for the production of APM foams. Using conventional, blowing-agent based powder compact melting type aluminum foams as reference, this study quantified the cost reduction potential of the aforementioned measures’ best combination as reaching nearly 50%. This was achieved with some effect on performance, namely a certain loss in average compressive strength, which was again compensated, however, by a reduction in scatter of this property. In engineering design, the latter would effectively alleviate the former, adverse, effect. Figure 2 illustrates the results based on data from the original study, showing, in the top image (Figure 2a), the relative contribution of different materials and processing steps not to the cost of the final foam, but to that of the foamable precursor material. In the bottom image (Figure 2b), again looking at the precursor material only, the yield of the different processes is added to the picture, and cost is shown relative to the conventional powder compact melting or Foaminal™ process, highlighting the level of reduction that can be achieved through the suggested measures [72].

Having thus discussed the background, as stated above, the primary aim of our own study is an attempt at linking cost and performance. We want to highlight which types of foams achieve superior properties in combination with attractive pricing. We will pursue this aim based on a classification of foams which distinguishes between various categories extending from highly stochastic over partially and fully ordered materials to those materials that are designed-to-purpose in terms of their cellular structure. We will discuss these categories in the following section, which also provides examples of major foam production processes associated with them. The following section will contrast the performance levels reached by foams originating from the various categories, and use this distinction as an ordering criterion. The final section will add the cost issue, and introduce first approaches for capturing cost of performance characteristics.

Naturally, the cost of performance must be matched not only with other types of cellular metals. Equally important is the question of how cellular metals compete with conventional materials which currently occupy the niches cellular structures might enter. These may include the respective cellular metals’ matrix materials in their non-porous, compact state.

## 2. Categorization of Foams and Associated Manufacturing Processes

Based on manufacturing methods, we introduce a classification system that differentiates these processes via the various levels of geometrical control. In it, we do not distinguish between foams and sponges but consider cellular metals of either kind.

**Figure 3:**
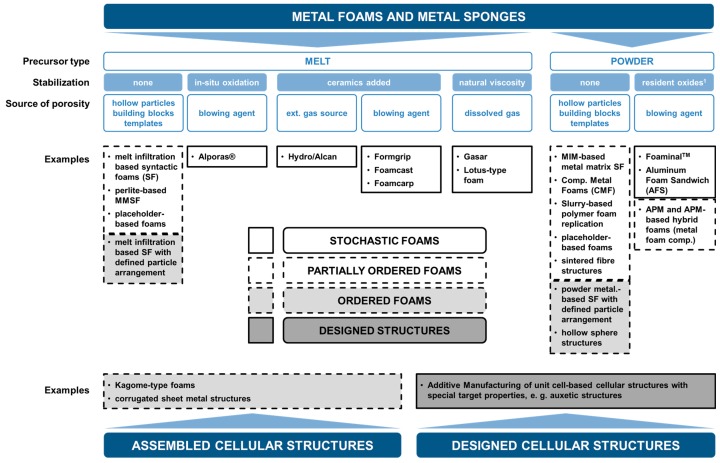
Relationship between a conventional classification scheme for metallic foams suggested by Banhart [76] and the approach proposed in the present study, which is based on the degree of order and additionally encompasses non-foamed types of cellular metals.

The following list, which is graphically illustrated in Figure 3, commences with methods and materials that are—from a generalized point of view—characterized by the least control over the pore shape, size, and spatial distribution and concludes with additive manufacturing methods that permit outstanding geometry control as the necessary basis for structures that are designed for specific performance characteristics, though often at a significantly increased cost. We structure this list of cellular metal manufacturing processes into four different categories, starting from stochastic over partially ordered and ordered to designed-to-purpose structures only made possible by additive manufacturing techniques:

- Stochastic Foams
  (a) Melt foaming using blowing agents, gas introduction (uncontrolled)
  (b) Powder metallurgy using blowing agents
- Partially Ordered Foams: Enhanced Structural Control on Pore Level
  (a) Melt foaming using controlled gas introduction
  (b) Melt foaming using solidification control
  (c) Placeholder methods
  (d) Precursor/replication methods
  (e) APM foams
  (f) Syntactic foams
  (g) Placeholder methods with ordered assembly of placeholders
  (h) Syntactic foams through ordered assembly of hollow particles
  (i) Metal fiber structures
- Ordered Foams: Ordered Assembly of Identical Structural Elements
  (a) Kagome-type foams
  (b) Corrugated sheet metal structures
  (c) UniPore structures
- Designed-to-Purpose Cellular Structures
  (a) Additive manufacturing approaches.

**Figure 4:**
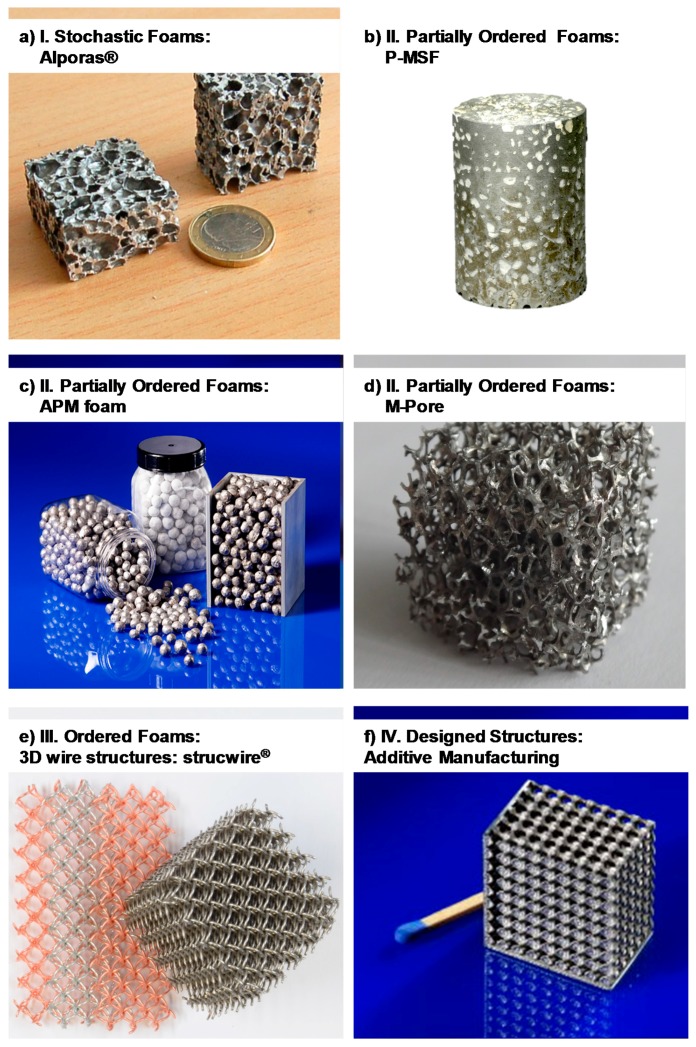
Exemplary images of foam samples representing the four main process categories, illustrating the levels of order and structural control associated with them [79]. (**a**) Alporas^®^ foam; (**b**) P-MSF foam; (**c**) APM foam; (**d**)M-Pore foam; (**e**) strucwire^®^ 3D wire structure; (**f**) additively manufactured 3D lattice structure.

**Figure 5:**
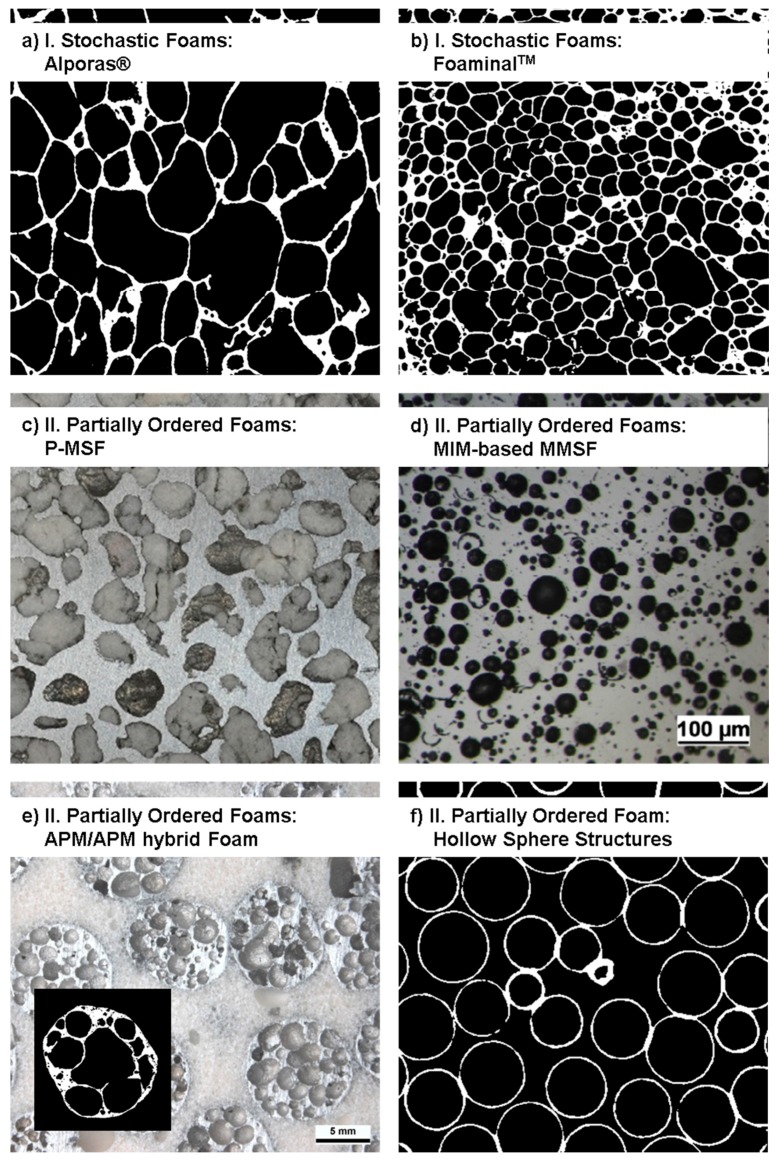
Exemplary images of the internal structure of specific types of foam representing the classes of stochastic (**a**,**b**) and partially ordered foams (**c**–**f**).

Figure 4 and Figure 5 depict foam structures representing these classes based on external images of samples and cross sections. The distinction between classes is not always as clear as it may seem—in reality, there are intermediate processes both between adjacent and more distant classes. One example of the first kind are syntactic foams created by infiltration of an ordered assembly of hollow spheres. An example of the latter kind are periodic or otherwise ordered APM foams [77], which consists of a controlled arrangement of so-called APM foam elements each of which exhibits internal porosity [45,78] originating from a powder metallurgical blowing-agent process [43,44]. Consequently, the hierarchy of structural control chosen as a basis of the present work is an approximation which allows for some deviation and may have to be revisited in follow-up studies. 

A comparison of performance-related cost must first of all bow to the impossibility of covering all materials, and thus effectively agree to make a choice. We have done this in an attempt to cover a representative cross-section of the available materials. To this end, our choice covers all the four classes of order introduced above. In the following section, we will briefly describe the processing of those materials we decided to focus on. For a broader overview of all the different materials and processes available, we refer the reader to works by Banhart [9,76] for the broad perspective, as well as Compton [42] and Kang [41] for the specific fields of AM and wire-based, assembled structures, respectively. 

### 2.1. Category I: Stochastic Foam—Alporas^®^, Foaminal™, and Others

Stochastic foams emerge from processes which by nature offer little or no direct means of controlling individual pore sizes, or the spatial arrangement of pores. Typical examples are techniques which employ blowing agents to induce pore nucleation and growth, i.e., processes that include a foaming step in the word’s original sense. Of these, variants exist which introduce the blowing agent in the molten matrix (Alporas^®^), while others rely on powder metallurgical production of a precursor material incorporating the blowing agent which is expanded through full or partial melting (Foaminal™ or powder compact melting process).

**Figure 6:**
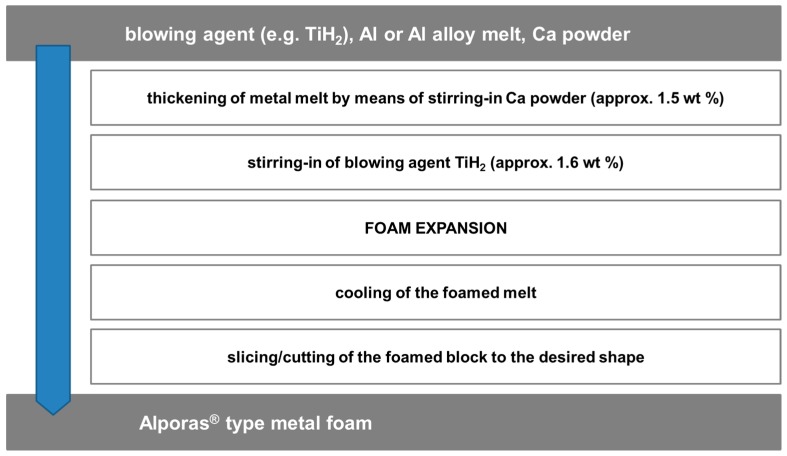
The main steps of the Alporas^®^ process.

Starting point of the Alporas^®^ process [27,28] as described in Figure 6 is an aluminum melt, the viscosity of which is modified through addition of Ca. The formation of Ca compounds with oxygen and/or aluminium is assumed to be the cause of this effect. Subsequently, the blowing agent (TiH_2_) is mixed into the thickened melt. Since the melt temperature of approximately 680 °C exceeds the lower end of the hydride’s decomposition range, foam expansion starts almost immediately. Some control over the expansion process is exerted by the application and maintenance of a constant pressure on the developing foam [76]. Density levels achieved thus can be as low as 0.2 g/cm^3^, which corresponds to porosity levels beyond 90%.

**Figure 7:**
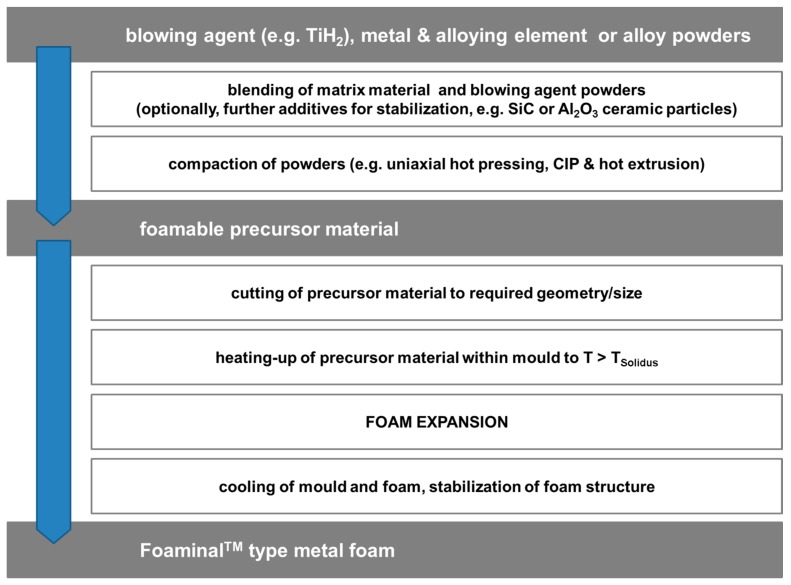
The main steps of the Foaminal™ or powder compact melting process.

In contrast to Alporas^®^, the Foaminal™ process [7,8,80] requires the matrix material to be present in powder form. Alloys can be formed either by using pre-alloyed powders or mixtures of elementary powders, or combinations of both [81]. In either case, a blowing agent has to be added to the powder mixture, which is then compacted to create a foamable precursor material. The most common blowing agent is TiH_2_ at content levels between 0.5 and 1 wt %. To better adapt their decomposition to the requirements of the process, blowing agents typically undergo a thermal treatment prior to mixing them with the matrix powders [22,23,82]. Consolidation itself must be performed in a way that ensures good welding between particles. This is typically achieved by choosing non-isostatic compaction processes, like hot extrusion, which generate high shear loads within the material, thus destroying particle surface oxide layers, creating new surfaces, and dispersing the oxides. Due to this intermediate step, the method is also designated powder compact melting process. The actual foaming step is performed in a closed mold at temperatures at least above the solidus, more commonly above the liquidus temperature of the matrix alloy. A special process variant includes so called aluminium-foam sandwich materials (AFS), which combine a core layer of foamable material with metallurgically bonded, conventional aluminium alloy face sheets. The build-up allows forming prior to foaming, yielding all-metal shaped sandwich materials [10,57]. The individual steps are graphically depicted in Figure 7 below.

Besides the Foaminal^TM^ or powder compact melting process, there is a number of related, solid state precursor material methods for production of metal foams. Some of these even use casting techniques in preparation of the precursor material, like the Formgrip or Foamcarp process [70,71].

While influencing size and shape of individual pores is not available, techniques like the modification of blowing agent decomposition, or of molten matrix properties like surface tension and viscosity, have been demonstrated to facilitate limited control at least of the pore size distribution. 

Further stochastic metal foaming processes use alternative paths for bubble nucleation. An example in this respect is the Hydro or Alcan™ process, which relies on injection of gas into a ceramic particle-stabilized (typical content levels approx. 10–20 vol %) aluminum melt by means of an impeller. The foam forms on the melt surface in this case, reaching a typical thickness of roughly 10–15 cm. For solidification, this foam layer is drawn off the melt surface using a conveyor belt and solidified. The process thus yields large foam panels with densities potentially even below those achieved via the Alporas^®^ process, with ranges given as 0.069 to 0.54 g/cm^3^ by Banhart [76].

Beyond characteristics of pores and porosity, each of the aforementioned processes is subject to certain types of defects that underline their stochastic character. On a global length scale, solidification of Foaminal™ type foams typically leads to a density gradient from core to center, culminating in the typical integral foam character produced in molds. Superimposed are effects of gravity, which lead to density gradients through mechanisms like drainage of liquid metal and buoyancy of pores. Drainage as such also increases the tendency towards cell wall rupture as a consequence of the resulting melt depletion within the cell walls. If rupture occurs, individual large pores are formed which broaden the cell wall distribution. Specifically in processes which start from a precursor material, large cells may also originate from cracks initiated in the material during intermediate stages of heating-up for foaming while the matrix is still in the solid state, but gas release from the blowing agent has already begun. In Foaminal™ foams, countermeasures against this effect include the aforementioned thermal modification of the blowing agent and/or selection of an adapted, low-melting matrix alloy.

On a length scale below that of individual pore size, emergence of cracks during solidification is not uncommon. Besides, cell wall thickness and grain diameter share the same order of magnitude, which may result in the existence of preferred failure sites. Similar effects may be attributed to corrugated cell walls [18,83].

Common matrices for foams of this kind are aluminum and zinc alloys, however, the PM processes have also been specifically adapted to processing of several other materials such as lead, zinc, iron and steel [84,85,86], or even gold [57]. Relative densities for aluminum foams are typically in a range between 0.2 and 0.35, while even lower levels have been achieved for Al foams when used as sandwich core, as well as for zinc or lead foams [85].

In terms of achievable mechanical properties, it may be noted that additives needed for stabilization of the liquid foam tend to influence failure mechanisms and strength. An example in this respect are the Ca additions needed for the production of Alporas^®^ foams, but sometimes also used in adapted powder compact melting processes [73]. Like ceramic particle additions, they result in increased brittleness of the foam, which usually causes a reduction of energy absorption in compression, even though initial yield and compressive strength have been shown to rise with balanced additions of suitably sized ceramic particles [21,25,26].

### 2.2. Category II: Partially Ordered Foams—Limited Structural Control on Pore and Spatial Distribution of Pore Levels

The synthesis of partially ordered foams allows some control over aspects like pore shape, pore size or size distribution, or spatial arrangement of pores. Only if full control is available over all of these aspects, we talk of ordered foams. Processes for production of partially ordered foams may thus provide handles to influence all the aforementioned characteristics to a limited degree, or at most all but one of them to the fullest. Typical examples of partially ordered foams are thus materials which integrate defined structural elements like porous or hollow spheres in an at least a partially stochastic manner in some kind of matrix. This general description matches many types of syntactic foams as well as the so-called APM foams, which will be described below.

Templating processes—which either use placeholders to create pores, or employ non-metallic foam precursors for geometry replication—offer the required geometrical command based on the fact that the typically polymeric templates themselves, which serve as either positive or negative images of the later metal foam, tend to be more susceptible to it.

Also part of the present category are foams which control the pore generation itself, rather than letting it happen. Solidification-controlled GASAR foams [29,30] are an example. A similar process, though with several variations, including the use of thermal decomposition reactions as primary source of hydrogen in contrast to the common high pressure method, has been studied by Nakajima et al. [31,33], and extended to a large variety of matrix alloys such as iron, cobalt, copper, silicon, magnesium and aluminium. Structures obtained include elongated pores, their axes coinciding with the direction of (unidirectional) solidification. In addition, local variation of pore shape and orientation within a single sample has been attained.

Some of these processes will be introduced in the following section.

**Figure 8:**
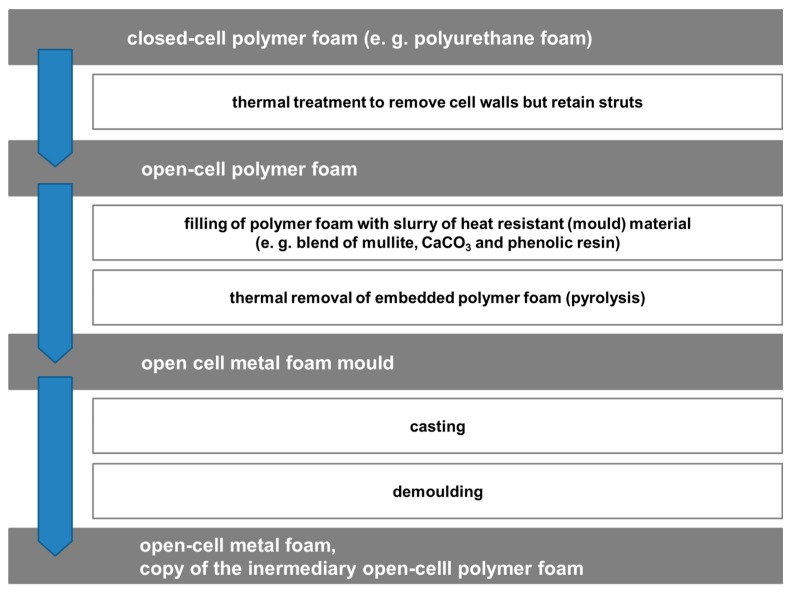
The main steps of the M-Pore process.

The M-Pore process detailed in Figure 8 is an example of a replication-based approach which uses polymer foams as a template to produce metal foams. To facilitate the replication, the original polymer foam is subjected to a thermal treatment meant to destroy cell walls but retain struts. The resulting open-cell polymer foam is then infiltrated with a slurry modelled on mold materials for investment casting. Drying of this slurry allows thermal removal of the polymeric phase. The result is a mold which is essentially a negative image of the polymer foam’s struts. Filling it with liquid metal and allowing it to solidify results in a metallic sponge which mirrors the reticulated polymer foam.

Besides the above method, other variants exist which realize the templating of the reticulated polymer foam in slightly different ways—examples include coating with metal particle based slurries and subsequent sintering, electroplating, or PVD metal deposition. 

Typical deficiencies of the M-Pore process which can lead to property degradation originate from the template. Geometry control depends on what was achieved in this respect in the polymer foam. The benefit in comparison to direct metal foaming techniques is that polymer foam evolution is, in principle, more accessible to any kind of control. Organic systems tend to lend themselves easier to modification of major parameters like viscosity or surface tension. Besides, processing temperatures are significantly lower. However, generally they, too, show random geometries (see e.g., PU foams). Besides, the pyrolysis process applied to remove the organic component is prone to leaving pores as well as residual carbon within struts.

**Figure 9:**
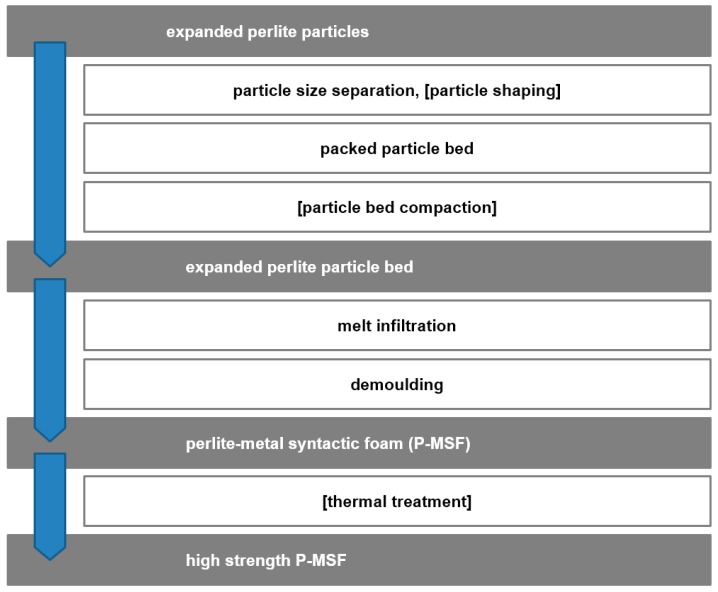
The main steps of the perlite metal matrix syntactic foam (P-MSF) process.

Perlite-based metal matrix syntactic foam (MMSF) or P-MSF is produced by melt infiltration of packed beds or porous particles (see Figure 9). It is an example for a wide range of MMSF that utilizes a variety of porous filler particles such as pumice, vermiculite [87], expanded clay [88], porous recycled glass [74], etc. The usage of filler particles permits a limited geometric control of the foam porosity. In general, two different porosity sizes must be considered, i.e., micro-porosity inside the porous particles and meso-porosity introduced by the particles within the metallic matrix. The usage of low-cost filler particles typically does not permit the control of micro-porosity. However, compared to the metallic matrix, the filler particles are usually weak and thus their micro-porosity is of limited importance for the macrosopic foam properties. In contrast, the meso-porosity (i.e., the space occupied by filler particles) defines the inverse geometry of the load-bearing metallic matrix and thus strongly affects both magnitude and scattering of the mechanical strength. In P-MSF, the meso-porosity has been successfully controlled by the size selection of filler particles [89,90], the filler particle shaping [91], and filler particle pre-compaction [92]. The spatial distribution of filler particles (and thus pore topology) is not completely random and governed by their positioning in packed particle beds. Gravity causes particles to settle within surface indentations between preceding particles. Vibration and/or compaction may be used to increase packing density. However, a direct control of particle (i.e., meso-pore) position is not possible. Packed particle beds are infiltrated with aluminum melt and demolded following solidification. Thermal treatment has shown to be an efficient mechanism to improve P-MSF strength [20]. Incomplete melt infiltration introduces a third type of porosity that tends to occur within the narrow channels close to filler particle contact points. These casting defects can act as seeds for macroscopic failure and should therefore be minimized. Possible strategies are the increase of infiltration pressure, superheating of the metallic melt, and the selection of melts with low shrinkage upon solidification. 

**Figure 10:**
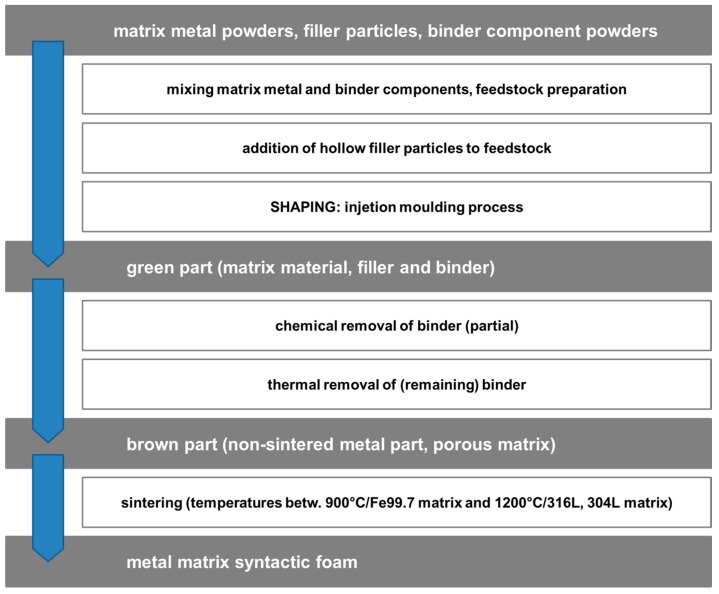
The main steps of the MIM process for production of metal matrix syntactic foams.

The P-MSF process is an example of a melt infiltration-based synthesis technique for syntactic foams. Metal powder injection molding (MIM) as presented in Figure 10 in contrast uses the matrix material in the form of a powder to which a polymeric binder, usually a mixture of thermoplastic polymers, wax, and lubricants is added at relatively high volume fractions. Mixing and kneading yields a feedstock which can be shaped in a manner very similar to plastic injection molding, using essentially the same equipment. Process temperatures and pressures are thus comparatively low, but the resulting ‘green part’ still contains a large amount of organics. These are removed chemically and thermally. The result is the brown part, which shows open porosity. Sintering leads to significant levels of shrinkage and yields metal parts approaching or even reaching the full theoretical density. The process adaptation which yields syntactic foams instead of solid metal parts is subtle. At the later stages of feedstock preparation, hollow glass or ceramic microspheres or cenospheres are added to the mixture, facilitating their even distribution within it. Given a suitable strength (typically 30 MPa isostatic compression strength and above) and small diameter (the extrusion process requires spheres to be smaller than approx. 100 µm in diameter), these microspheres retain their integrity throughout the process and thus provide the envisaged levels of porosity. Furthermore, as has been shown in comparison with non-syntactic metal foams of identical matrix, they contribute notably to compression strength. Material development is currently focused on iron [35,93] and steel grades like 316 L or 304 L [36,94]. Besides, Invar matrix materials have been produced [37,95,96].

The shape and size of pores can be closely controlled; however, their location is most often random. Some manufacturing procedures permit a limited control of the topology of the added particles, but these are typically of experimental or lab-scale character and aimed at improved understanding of material performance rather than commercial or even mass production.

Typical defects in syntactic foams depend on the production process. In infiltration-based processes, porosity may originate from insufficient filling of the inter-particle voids or entrapped gases—introduced through turbulences—or result from solidification shrinkage.

Residual porosity may also be observed in PM syntactic foams. Materials produced via the metal powder injection molding or MIM process are characterized by binder content levels which are higher than in other powder metallurgical processes, and can reach up to 50%. Effectively, this means that a corresponding level of sinter shrinkage is necessary to achieve a fully dense metallic part. In entirely metallic materials, this is easily achieved, however, in syntactic foams, the embedded microspheres may hinder homogeneous shrinkage, thus leading to potentially higher levels of residual porosity than observed in non-syntactic material variants under otherwise identical conditions. MIM production of iron and steel matrix syntactic foams using hollow glass microspheres as filler materials somewhat alleviates this effect, since the microspheres experience softening from 600 °C, while sintering temperatures range between 900 °C (pure iron/Fe99.7, see [35,93])/1000 °C (Invar, see [95,96]) and 1200 °C (steel grades like 316 L [94]). In contrast, the use of cenospheres, with thermal stability maintained up to temperatures of 1400 °C according to specification, pose more of a problem in this respect due to the obvious lack of softening [37].

For a different type of syntactic foams, the so-called composite metal foams as developed by Neville and Rabiei based on metallic hollow spheres [97,98,99], the adverse effect of hollow spheres on matrix porosity levels is believed to be somewhat alleviated by the expansion of gases within the hollow spheres, through which a certain level of pressure is exerted on the matrix between spheres during sintering. Densification is assumed to be supported by this effect.

Further types of cellular metals which are closely related to syntactic foams are hollow sphere structures, which do not embed hollow particles in a matrix, but join them to each other. This can either be done directly, e.g., in parallel with the consolidation through sintering of powder metallurgically produced metallic hollow sphere shell materials, or in a subsequent processing step involving some bonding agent. Structures of the latter kind have, for example, been discussed by Fiedler et al. [100]. A more recent approach uses spark plasma sintering (SPS) to join cenospheres sputter-coated with metallic materials like copper [101].

**Figure 11:**
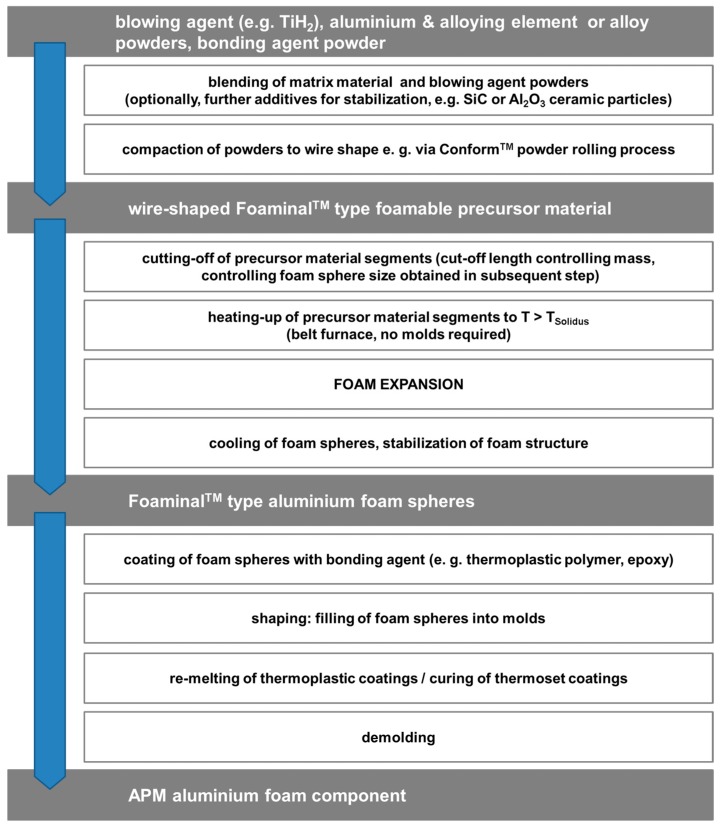
The main process steps of the APM process described in relation to the aforementioned Foaminal™ process.

The so-called APM foams are also similar to this approach: Structurally, they resemble syntactic foams in incorporating spherical particles. These, however, are of mm rather than µm size and as in the case of hollow sphere structures, not surrounded by a solid matrix, but joined to each other via a surface coating. Besides, they link APM foams to the Foaminal™ process, since it is essentially this type of material the characteristic spheres are made of. The connection is reflected in Figure 11, which details the manufacturing process. The advantage of Conform™ as consolidation process in precursor material production is the direct path from powder to wire this method offers. Cutting off segments of this wire and passing them through a belt furnace will lead them to form spheres, which can be coated with thermoplastic polymers as well as epoxies. Filling these separate, coated spheres into a mold and activating the coating (i.e., melting or curing it) will lead to shaped parts which are not subject to the size limitations of Foaminal™ materials. The addition of a blowing agent to the organic coating prior to applying it to the spheres will create an expandable surface layer capable of filling the cavities in between the APM foam spheres. The result is a hybrid polymer-metal foam.

In terms of the scatter of properties, APM foams share the possibility of local variations in the spatial arrangement of the spheres with random hollow sphere structures [46,102]. Weak spots induced thus can in principle be starting points for tensile failure, or initiate and support the development and propagation of deformation bands under compressive load. APM foams may be assumed to be more susceptible to such effects since they exhibit a wider distribution of deviations from the ideal spherical shape. Besides, their internal structuring, which partly reflects the stochastic nature of the Foaminal™ process, will lead to a larger variation of properties of individual spheres of identical nominal size and density in contrast to otherwise comparable metal hollow spheres. For all cellular structures relying on the connection of spheres, the small contact area between the spheres, which may be somewhat increased by an adhesive, may be a weakness specifically if tensile loads common to the inter-sphere joint’s plane are applied.

Beyond those described in more detail here, further types of partially ordered foams include metal fiber structures as described by Veyhl et al. in terms of their thermal properties [103] and by both Veyhl et al. and Andersen et al. with respect to mechanical characteristics [104,105]. Production of large numbers of fibers is usually done by melt spinning, while the connection between the fibers is achieved through sintering processes. The result is a structure that resembles a metallic felt. Its assumed partial order is based on the available control of fiber characteristics. Besides, the arrangement of fibers is normally not completely random, specifically if sheet-like structures are produced, in which the fibers’ longitudinal direction tends to be parallel to the sheet’s center plane.

### 2.3. Category III: Ordered Foams—Ordered Assemblies of Identical Structural Elements

Cellular metals that originate from an assembly of standardized structural elements can either be based on zero-, one-, or two-dimensional examples of such building blocks: Hollow spheres might in this sense be considered point-like, while the wires that form Kagome-type structures [106] are essentially one-dimensional, and corrugated sheet metals joined to each other represent an originally 2D structure. The assembly of structural elements enables a high uniformity of the resulting foam geometry without significant structural defects. As a result, more predictable material properties can be expected. However, the range of possible foam geometries is of course limited by the shapes of their building blocks.

The application of weaving techniques has proven to be an efficient approach to manufacture open-celled metallic foams with close control of truss geometry [107]. To this end, helically-formed wires are systematically assembled to form periodic three-dimensional structures. Individual wires are then joined using brazing, soldering, sintering, or adhesive bonding. Additional control of foam geometry can be introduced by the controlled variation of wire thickness [108]. Another interesting approach is the combination of different types of structural elements such as helically-formed wires in conjunction with metallic hollow spheres [109]. 

Because of the lack of an actual foaming step, ordered cellular metals created by a combination of exactly-defined building blocks do not suffer from cell wall rupture, drainage, or similar effects associated with this step.

### 2.4. Category IV: Designed-to-Purpose Cellular Structures

Additive manufacturing techniques have opened up new possibilities for the production of cellular metals. For the first time, these techniques offer a high degree of geometrical flexibility in the realization of such structures. The basis of these vastly enhanced capabilities is the way components are built up—usually this is done layer by layer, and typically, this approach gives access to any single volume element, all of which together form the material. This voxel-by-voxel fusing of the material to the already built part of the sample in direct translation of a digital model is usually done layer by layer. This setup can create basically any geometry, including internal cavities, at a resolution matching the voxel size as defined by the layer thickness and the area resolution of the consolidfation/fusion process—in Laser Beam Melting (LBM), for example, the latter would roughly equal the size of the melt pool produced by the laser during scanning of the current layer of metal powder. Geometric limitations may be introduced by the selected manufacturing technique. As an example, neither EBM nor LBM permit the manufacturing of empty closed pores because metallic powder becomes entrapped within closed cavities. Other methods based on melt extrusion only permit a limited material overhang between subsequent layers. The angle of overhang can be increased by printing support structures, which are normally removed once printing is completed. However, these support structures become a permanent component of the printed material if located within closed pores. As a result, designed cellular structures typically exhibit open pores.

**Figure 12:**
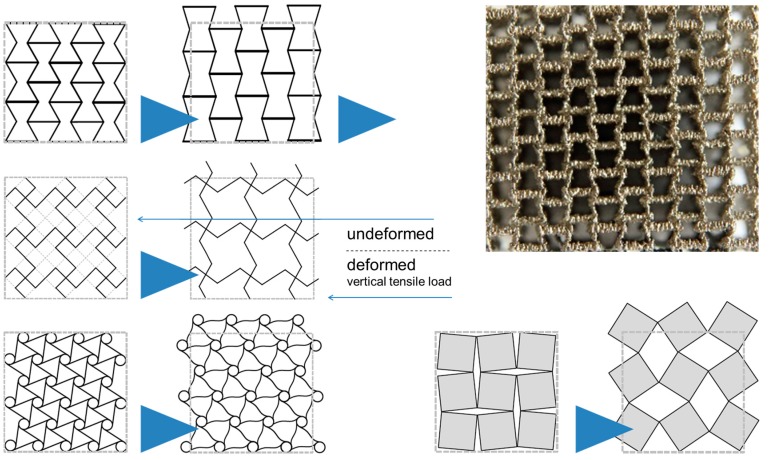
2D sketches of structures showing auxetic behavior and example of a physical realization of one of them, a re-entrant structure produced by means of additive manufacturing techniques [111].

Besides optimization of general mechanical behavior in terms of properties—like stiffness, strength, and energy absorption—this new flexibility provides the opportunity to design specific mechanical characteristics unknown in solid materials. Auxetic behavior, i.e., the furnishing of such structures with a negative Poisson’s ratio, is a prominent example in this respect which is illustrated in Figure 12. Further aspects in this respect include mechanical damping, which, as Warmuth and Körner have pointed out, can be tuned towards the behavior of a phononic bandgap material—a filter fading out specific vibration frequencies [110]. In additive manufacturing of cellular metals, simply increasing the level of mechanical damping is thus often replaced by an attempt to tailor it towards optimum selectivity. Auxetic structures and their likes thus add a further dimension to the property fields that—as depicted in Figure 1—are typically associated with metal foams.

However, besides the aforementioned developments towards specialized properties, additive manufacturing has also been used to optimize the more conventional mechanical properties of cellular materials. For this purpose, several studies have looked at different types of unit cells that are then repeated within samples and components produced. The typical lower limit of feature sizes is in the range of a few 100 µm for the most common industrial AM processes used in production of metallic parts, like Laser Beam Melting (LBM) or Electron Beam Melting (EBM).

Among the problems additive manufacturing of cellular structures faces, cost is a major one. Typically, AM processes can massively decrease lead times, as no tools need to be constructed. However, for medium to large scale series, this advantage is counterbalanced by the relatively low productivity. Thus for large production runs, where costs of tooling can also be spread over a large number of parts, AM is typically less competitive unless secondary advantages like the elimination of assembly processes can be achieved by integrating several components into a single one. Besides, the metal powders certified for additive manufacturing are cost-intensive.

In terms of properties, several studies have shown that materials consolidated by means of AM processes can reach the performance levels of e.g., cast parts—or even exceed them. A critical aspect, however, is the limited surface quality of the parts, which can result in curtailing of mechanical property values. Besides, the inhomogeneous heat supply and dissipation esperienced by AM parts caused by—depending on the process—the layer- and pointwise introduction of thermal energy will almost inevitably cause residual stresses in the material.

## 3. Mechanical Performance

**Figure 13:**
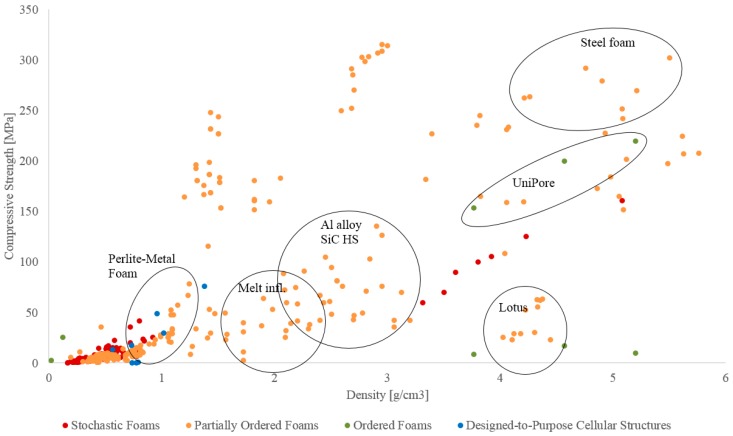
Comparison of the compressive strength of different types of cellular metals over a wide density range.

**Figure 14:**
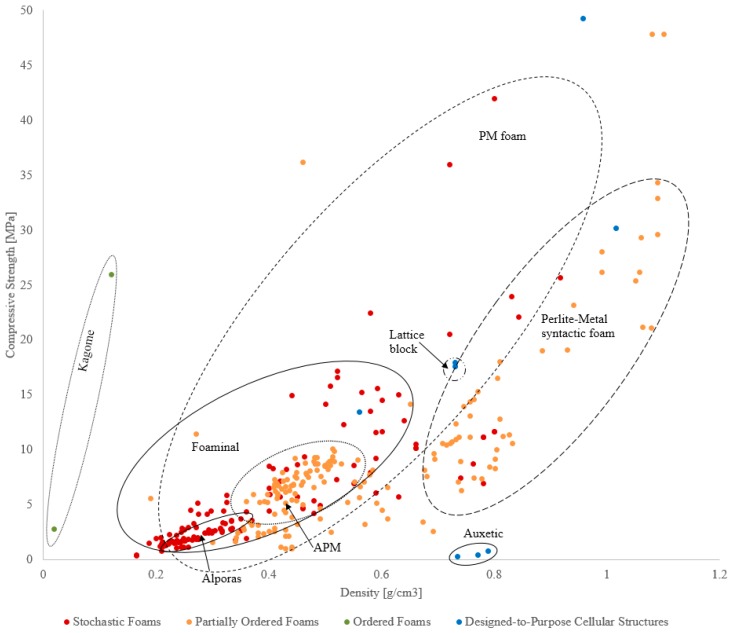
Comparison of the compressive strength of different types of cellular metals, detailed view of the lower density range included in Figure 13.

**Figure 15:**
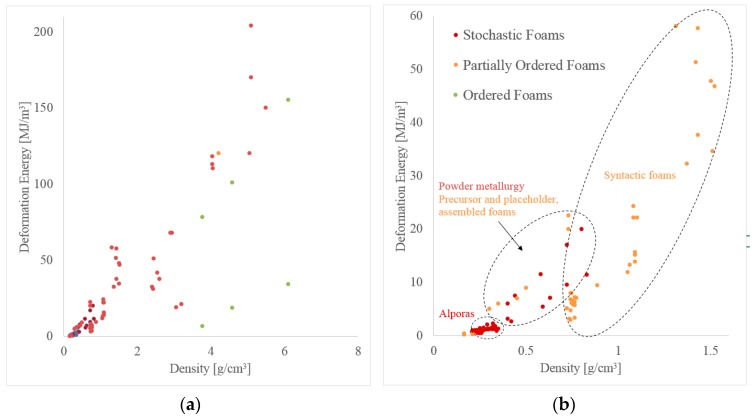
Comparison of the energy absorption capabilities of different types of cellular metals, (**a**) covering a density range from 0 to approximately 6 g/cm^3^, (**b**) showing the low density range between 0 and 1.6 g/cm^3^ in more detail.

**Figure 16:**
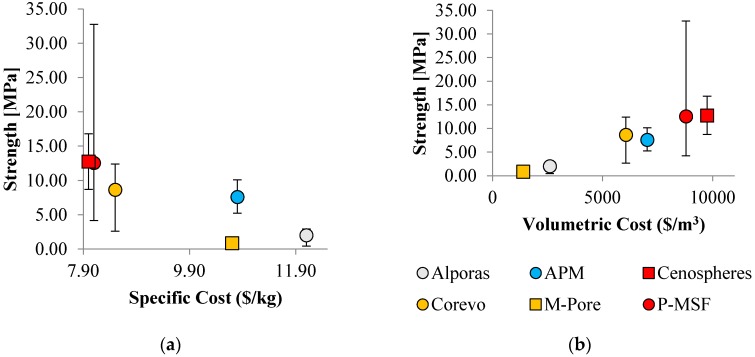
Material strength plotted versus (**a**) specific cost and (**b**) volumetric cost for selected types of cellular metals.

The following comparison of typical performance indicators per type of foam is rooted in mechanical characteristics, as for this class of properties, by far the most comprehensive database is available in the published literature. Readers should note that the quantitative data represented in Figure 13, Figure 14, Figure 15 and Figure 16, as well as associated cost information wherever available, is provided in the Appendix in Table A1, Table A2, Table A3 and Table A4.

### 3.1. Compressive Strength

The section focuses on compressive performance of different types of cellular metals. The compressive data collected from the literature is given in Figure 13. Since the data shown in the figure represents the findings of an extensive literature overview, the expression ‘compression strength’ accounts in some cases also for yield stress and plateau stress values, depending on the availability of the data. The given values including the references are gathered in the Appendix Table A1, Table A2, Table A3 and Table A4. Data points are color coded according to the categories introduced above. 

The stochastic foams fabricated by melt foaming and powder metallurgy, the partially ordered foams and designed-to-purpose structures are predominantly low density cellular materials. The syntactic foams and foams fabricated by precursor and placeholder methods (partially ordered foams) exhibit higher densities. It must be noted that the diagram includes cellular metals fabricated from different base materials (Al, Ti, and their alloys, as well as steel, iron, copper), a fact which greatly influences the obtainable absolute density range. Nevertheless, even with this reservation in mind, it can be observed that the syntactic in contrast to the conventional foams cover a wide range of densities as well as compressive properties, providing them with an exceptionally large design space. Needless to say, this freedom of design is linked to the additional property-controlling handles offered by these materials, which not only include matrix material and porosity, plus, on a secondary level, geometrical aspects of the latter, but also features of the hollow particles like particle density, shell material or shell thickness-to-diameter ratio.

Figure 14 gives a more detailed insight into the low-density cellular metals fabricated from lightweight metals and their alloys (i.e., Al, Ti). The melt foaming group (stochastic foams) is limited to Alporas^®^ which shows slightly lower strength compared to other materials with similar density. Stochastic foams fabricated by powder metallurgy have a wider density range and exhibit a superior strength-to-weight performance. Partially ordered foams fabricated by precursor and placeholder methods are gathered in a narrow band with a lower strength to density ratio. The syntactic foams can be found at higher densities (>700 g/cm^3^) and for a given density other materials exhibit higher strength. However, they extend the design space to high absolute strength values at very high densities (Figure 13). The ordered foams are limited to Kagome which shows very good compressive performance. It should be noted that the superior performance is to a great part induced by the base material (wrought Ti alloy). The designed-to-purpose cellular structures are grouped in the density band from 0.2 to 0.8 g/cm^3^. In general, they show slightly inferior performance in comparison to the foams fabricated by powder metallurgy and similar performance to lightweight syntactic foams. On the other hand, low compressive strength of e.g., the auxetic materials cannot be interpreted as a weakness as these materials have a very specific design focus which clearly deviates from optimum mechanical performance as expressed by strength-to-weight ratios and similar definitions.

### 3.2. Energy Absorption

In the following, the energy absorption capacity of cellular metals is discussed with respect to their density. Metallic foams can be used for impact protection in transportation where a combination of low weight (and thus energy consumption) and high energy-absorption is beneficial. As previously shown in Figure 13 and Figure 14, it is well established that the strength of cellular materials increases with density. Deformation energy is the integral of stress over strain and thus increases with strength. Accordingly, a similar trend emerges in Figure 15 where high-density foams exhibit distinctly higher deformation energy. 

Compared to material strength, less data could be found in the literature on energy absorption characteristics. In addition, the data points shown are slightly inconsistent as the upper strain limit chosen by the various authors for integration of stress-strain-curves differs between studies. The most common criteria for this strain limit are 50% engineering strain, densification strain, or the testing machine load limit. Two different plots are shown in Figure 15 below where the left one covers the complete density range and the right one focuses on lightweight foams with densities below 1.6 g/cm^3^.

Only data for one material produced using melt foaming can be shown in the plot (Alporas^®^, stochastic foam category). Due to the low density of the material, a low deformation energy is observed. Powder metallurgy foams (stochastic foam) also fall within the low-density range. They exhibit a good specific energy absorption efficiency, i.e., show a high deformation energy in relation to their density. The partially ordered foams (precursor and placeholder foams as well as assembled foams) exhibit similar performance. As in the case of compressive strength, syntactic foams cover a wider range of deformation energy and density. At low density (i.e., ≲1 g/cm^3^) they exhibit a low specific deformation energies and are outperformed by the other foam types. However, their performance rapidly increases with density where they exhibit the highest ratio of deformation energy to density. It should be mentioned that some of these data points are obtained by integration up to the maximum test load rather than a strain limit defined e.g. in relation to sample porosity, like densification strain, and may thus over-predict the deformation energy of these materials.

In comparison to the strength plots shown in Figure 13 and Figure 14, less scattering of data points is observed for energy absorption. This becomes visible as a close correlation between energy absorption and foam density. In comparison, material strength exhibits a higher sensitivity towards the foam manufacturing method, i.e., for the same density vastly different material strengths can be observed. A possible explanation is the onset of plastic deformation from local material defects, which therefore determine the macroscopic material strength. In contrast, energy absorption averages material strength over a large deformation range and the impact of initial material defects diminishes.

## 4. Cost of Performance

### 4.1. General Approach

The aim of the following section is the evaluation of material performance (i.e., material strength and energy absorption) relative to material cost. As discussed earlier, this approach requires careful interpretation. For example, outstanding material strength may justify a very high cost for a given application even if a lower ratio of performance-to-cost exists. A second limitation is the lack of commercialized metallic foams and the associated difficulty in estimating cost. As a result, only a small subgroup of cellular metals is considered.

In general, costs arise for raw materials, processing, and post-processing. Processing cost is determined by various factors, most importantly infrastructure, consumables, energy, personnel, and maintenance. Exploring the cost of a material detached from a concrete production scenario is a difficult task. For this reason, we have based our comparison on the assumption that with increasing lot sizes, specific aspects of cost will see a reduction of their relative importance. This is, for example, the case for infrastructure costs, but to a certain degree also for personnel. Assuming that increasing the lot size allows for investment in process automation, the contribution of which will once again decrease on a per kilogram basis the larger the production volume. In addition, the cost for post processing (e.g., cutting, bonding, machining) strongly depends on the particular application and thus cannot be readily included in this comparison. 

As a result, only costs that arise for all applications and do not reduce with quantity are considered. These costs are for raw materials (e.g., metallic powders and foaming agents), consumables (e.g., templates or space holders), and energy (required for casting or sintering). The procedure is explained on the example of Perlite Metal Syntactic Foam (P-MSF) and the results for all materials are summarized in Table 1 below.

P-MSF is manufactured by the combination of expanded perlite particles with melt aluminum. Typical volume fractions of aluminum (Al) and perlite (Pe) are $\phi_{Al}=$ 40% and $\phi_{Pe}=$ 60%, respectively. The cost for aluminum (A356—standard casting alloy) is estimated at USD 8 $/kg (USD 21,600 $/m^3^) and typical cost for expanded perlite is USD 0.1 $/kg (USD 120 $/m^3^). In the case of P-MSF no costs $C_{FA}$ for a foaming agent or consumables $C_{Cm}$ arise. Considering a starting temperature $T_{0}=$ 300 K, aluminium melting temperature $T_{M}=$ 1023 K, density $\rho_{Al}=$ 2700 kg/m^3^, specific heat capacity $C_{Al}=$ 0.91 kJ/kg.K, and latent heat Δh= 339 kJ/kg a total melting energy of 0.259 kWh/kg is required. Multiplication with an assumed energy price of 0.1 $/kWh yields the approximate energy cost $C_{E}$. Accordingly, the volumetric cost $C_{V}$ of P-MSF can be calculated using

(1) $$C_{V}=\underset{Aluminium\;cost}{\underbrace{\phi_{Al}\cdot 21600\frac{\$}{m^{3}}}}+\underset{\begin{array}{c} Filler\;particle\;\\ cost\;C_{FP} \end{array}}{\underbrace{\phi_{Pe}\cdot 200\frac{\$}{m^{3}}}}+\underset{Energy\;cost\;C_{E}}{\underbrace{\phi_{Al}\cdot \rho_{Al}\cdot \left[C_{Al}\cdot \left(T_{M}-T_{0}\right)+\Delta h\right]\cdot 0.1\frac{\$}{kWh}}}+C_{FA}+C_{Cm}=8790\frac{\$}{m^{3}}$$

The specific cost $C_{m}$ of P-MSF is obtained by the division with the material density (in this case P-MSF, $\rho_{P-MSF}=$ 1080 kg/m^3^)

(2) $$C_{m}=\frac{C_{V}}{\rho_{P-MSF}}=8.1\frac{\$}{kg}$$

Typically, the analysis of volumetric cost favors materials with high porosity. This is due to the relatively high cost of aluminum which decreases if the majority of the material volume is occupied by pores and/or low-cost filler particles. Conversely, property- or performance-specific cost favours materials with low porosity. In this case, the superior mechanical characteristics of low porosity materials outweigh a marginal cost reduction per unit mass. Pores and filler particles exhibit negligible mass and therefore the specific cost of a metallic foam is similar to the specific cost of aluminum (i.e., USD 8 $/kg).

Considering the above equation in view of MIM-based syntactic foams using hollow glass microspheres or cenospheres as filler, besides the matrix metal costs (corresponding to the aluminium cost in the equation), the cost of the organic binder materials have to be taken into account. However, when seen from the application perspective of a solid component with limited mechanical performance requirements which are met both by the solid material and the associated syntactic foam, production of the latter basically translates into a replacement of metal powders suited for the MIM process by hollow filler particles. Since the price of the latter is at least one order of magnitude lower than that of the metal powders, a substantial cost reduction can directly be obtained. Examples in this respect include e.g., Fe99.7, Invar or 316L-based PM syntactic foams basing porosity on glass microspheres or cenospheres [35,36,37,93,94,95,96].

### 4.2. Strength and Cost

Figure 16 below shows material strength relative to cost. Error bars indicate minimum and maximum strength of the material and the average value is shown by a marker. P-MSF exhibits the widest variation of strength as materials of different densities are considered. However, it should be mentioned here that, in all cases, only the average density is considered for cost evaluation and thus no error bars are shown for the x-direction. 

In the figure on the left, specific cost is considered. Data points with optimum performance are located in the top left corner of the plot. It is apparent that syntactic foams exhibit superior performance exhibiting maximum strength at the lowest specific cost. The explanation is their relatively low porosity results in superior material strength. In addition, the mass of the material is predominantly made up by the aluminum phase and only a small fraction of inexpensive filler particles (expanded perlite or cenospheres) are added. As a result, the specific material cost is similar to the selected aluminum alloy. Precursor and placeholder materials exhibit a slightly elevated specific cost. The main reason is their increased porosity resulting in a higher mass fraction of required precursors. In combination with inferior mechanical properties, this results in a decreased specific cost performance. The specific cost performance of APM foam and Alporas^®^ is poor due to the added requirement for an expensive foaming agent (which does not contribute towards the foam mass).

A different picture emerges when considering volumetric cost (see figure on the right). In this case, an approximately linear trend can be observed. Materials will high porosity exhibit the lowest volumetric cost. The explanation is the decreased mass of aluminum per volume. M-Pore exhibits the lowest volumetric cost (p≈95%), followed by Alporas^®^ (p≈92%) and Corevo (p≈74%). In contrast, syntactic foams with low porosity (p<60%) exhibit a significantly higher volumetric cost. Due to the increase of strength with decreasing porosity the linear trend emerges. Manufacturing technology causes minor deviations, i.e., Corevo has a higher volumetric cost efficiency compared to APM. In this case, the explanation is the lower cost of the salt dough precursor compared to the foaming agent required for the APM production. 

### 4.3. Energy Absorption and Cost

**Figure 17:**
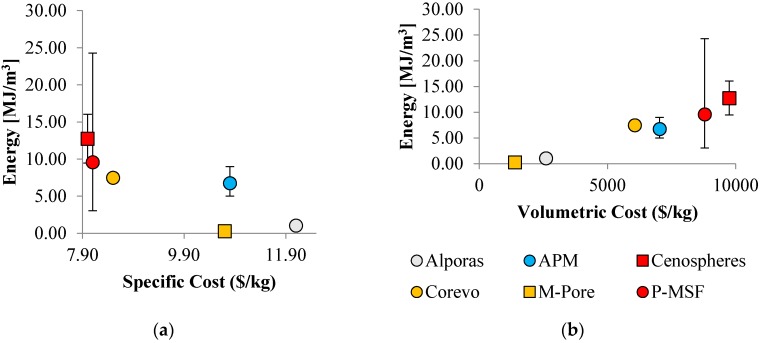
Absorbed energy plotted versus (**a**) specific cost and (**b**) volumetric cost.

Figure 17 shows the absorbed energy plotted versus specific and volumetric cost. Comparing these graphs to Figure 16, similar trends emerge. This can be explained by the close correlation between material strength and plateau stress. The absorbed energy is the integral under the stress strain curve and thus closely related to the plateau stress. As a result, low porosity materials again exhibit superior specific cost performance whereas high porosity foams exhibit a lower volumetric cost.

## 5. Conclusions

Having summarized the data above, the question we remain faced with is: “Is there is a sweet spot—i.e., a best compromise—between cost and performance?” The answer is that this will very likely be different for ‘bulk’ (e.g., roadside barriers) and ‘high-end’ applications (aviation).

No application is controlled by just one performance indicator, and all applications differ in terms of the monetary benefit of performance. As has been pointed out above, weight savings in automotive and aerospace applications differ greatly in this respect. Though there may be an independent figure describing cost of performance, there is no such figure to denote its value unless a link to a specific application can be established.

In our comparison, designed structures produced by means of additive manufacturing methods turn out to be among the most costly material variants, even if we relate their cost to their often outstanding performance. This outcome of our survey deserves further scrutiny. For one thing, there clearly are applications that justify this cost level, and since the AM material variants dominate both in price and performance, wherever top levels of the latter are a must, these materials will find their applications. There is, however, another aspect which also concerns other materials—it is the step from material to product. Some of the processes we have looked at only yield products of very simple geometry that need to be further processed for integration in an actual engineering component. The classical Alporas^®^ process is one example in this respect. Other processes can yield foam parts of very complex shapes—to a certain degree, the powder compact melting process is among these, and to a much higher degree the MIM process for producing syntactic foams. The additive manufacturing process marks the other end of the spectrum, with highest geometric complexity possible both on the length scale of the cellular structure and of the final part. and allowing arbitrary transitions between porous and solid regions within the same part. 

Even further to this aspect, it must be mentioned that the geometrical freedom accessible to the designer of an AM part can be utilized to design structures with unique properties. Three dimensional auxetic structures may be considered in this respect [112,113,114], or, given that multi-material AM processes are available, structures exhibiting tailored positive or negative coefficients of thermal expansion as suggested by Lakes [115], or phononic bandgap materials as recently presented by Warmuth et al. [110,116]. Naturally, neither of these materials can fairly be judged based on strength and energy absorption.

## Figures and Tables

**Table 1:** Overview of cost figures for selected types of cellular metals and their base materials.

Property	Alporas	APM	Cenospheres	Corevo	M.Pore	P-MSF
$\phi_{Al}$ (%)	8	25	45	25	5	40
$C_{FA}$ ($/m^3^)	871	819	-			-
$C_{Fp}$ ($/m)	-	-	free			120
$C_{Cm}$ ($/m)	-	-	-	320	100	-
$C_{E}$ ($/m)	6.0	18	33.6	19.8	3.7	29.9
$C_{V}$ ($/m)	2600	7030	9750	6060	1390	8790
ρ (kg/m)	216	650	1215	715	130	1080
$C_{m}$ ($/k)	12.1	10.8	8.0	8.5	10.7	8.1

## Appendix A

**Table A1:** Stochastic foams’ properties and cost.

Density	Metal (Matrix)	Comp. Strength (C), Yield Stress (Y) or Plateau Stress (P)	Energy Density	Specific Cost Estimate	Volumetric Cost Estimate	Reference
(g/cm^3^)	(-)	(MPa)	(MJ/m^3^)	(EUR/kg)	(EUR/m^3^)	
0.17–2.1	Al	0.5–0.9 (P)	0.22–0.34	10.7	1390	[117,118]
0.19–0.84	Al	1.5–22.1 (C)				[24]
0.2–0.8	Al	2–7 (Y)				[24]
0.21–0.33	Al		0.71–1.33	12.1	2600	[119]
0.25–0.34	Al	1.8–3 (P)	0.93–1.55	10.3	5770	[54]
0.26–0.27	Al		1.05–1.09			[120]
0.26–0.59	Al	3.4–15.6 (C)				[24]
0.29–0.4	Al	2.7–8.6 (P)	2.12–6.07			[54,60]
0.35–0.66	Al	3.8–10.6 (C)				[121]
0.42–0.83	Al	5.8–24 (P)	2.66–11.45			[60,65]
0.44–0.8	Al	15–42 (P)	7.5–20			[122]
0.6	Al	11.7–14.6 (C)				[17]
0.66–0.8	Al	10.2–11.7 (P)				[123]
3.31–5.08	Steel	60.1–161.3 (C)				[124]

**Table A2:** Partially ordered foams’ properties and costs.

Density	Metal (Matrix)	Comp. Strength (C), Yield Stress (Y), or Plateau Stress (P)	Energy Density	Specific Cost Estimate	Volumetric Cost Estimate	Reference
(g/cm^3^)	(-)	(MPa)	(MJ/m^3^)	(EUR/kg)	(EUR/m^3^)	
0.19–0.46	Al	0.3–12 (Y)				[104]
0.3–0.5	Al	6.2 (P)	5–9			[43]
0.3–0.6	Steel	1.7–4.6 (P)				[125]
0.34–0.74	Al	2.5–12.7 (C)				[44,121]
0.41–1.08	Al	5.6–26.2 (C)				[126]
0.43–0.8	Al	1.2–11.4 (P)				[105]
0.67–0.77	Al	3.5–12.4 (Y)	7.2–8	8.5	6060	[127]
0.74–0.76	Al	7.5–8.8 (P)	4.8–5.7	10.8	7030	[102]
0.74–1.08	Al	7.1–52.6 (P)	3.2–24.3	8.1	8790	[92]
1.05–1.09	Al	25.4–34.4 (P)	11.9–15.6			[38]
1.31–1.52	Al	154–248 (C)	32.3–58.1			[128]
1.56–4.76	Steel	50–292 (Y)				[129]
1.72–2.5	Al	2.8–48.6 (C)				[130]
1.82	Al	152–181 (C)				[131]
2.4	Al	67 (P)	32.3			[132]
2.43–2.45	Steel	60–105 (P)	31–35			[133]
2.55–3.06	Fe	42.3–81.4 (C)				[98]
2.55–3.06	Steel	36.2–127 (P)	18.9–67.8			[98]
2.6–3.2	Steel	42.3–136 (P)	21–68			[132]
2.95	Steel	76 (C)				[98]
3.34–5.21	Fe	182–270 (Y)				[93]
3.39–4.93	Fe	227–231.3 (C)				[94]
3.79–5.01	Fe	235.4–262.8 (C)				[94]
4.02–4.44	Steel	25.5–63.3 (C)				[134]
4.06–5.05	Fe	159–165 (Y)	110–120			[95]
4.21–5.49	Invar	160–198 (Y)	113–204			[95]
4.86–5.76	Steel	173.2–208.2 (C)				[36]
4.9–6	Steel	279.5–607.5 (Y)				[94]

**Table A3:** Ordered foams’ properties and costs.

Density	Metal (Matrix)	Comp. Strength (C), Yield Stress (Y), or Plateau Stress (P)	Energy Density	Specific Cost Estimate	Volumetric Cost Estimate	Reference
(g/cm^3^)	(-)	(MPa)	(MJ/m^3^)	(EUR/kg)	(EUR/m^3^)	
0.018	Ti	2.8 (Y)				[106]
0.12	Ti	26 (Y)				[106]
3.76–4.57	Cu	153.6–200.1 (P)	78.2–100.9			[50,52]

**Table A4:** Designed-to-purpose cellular structures’ properties and costs.

Density	Metal (Matrix)	Comp. Strength (C), Yield Stress (Y), or Plateau Stress (P)	Energy Density	Specific Cost Estimate	Volumetric Cost Estimate	Reference
(g/cm^3^)	(-)	(MPa)	(MJ/m^3^)	(EUR/kg)	(EUR/m^3^)	
0.56–1.34	Ti	13.5–76.3 (Y)				[135]
0.73	Al	8 (C)				[136]
0.74–0.79	Cu	0.3–0.9 (C)				[137]
